# Antimicrobial and Anti-Inflammatory Bioactive Peptides: Their Role in Potential Therapeutic Applications for Periodontitis—A Narrative Review

**DOI:** 10.3390/nu17193105

**Published:** 2025-09-30

**Authors:** Federica Tonolo, Renata Cristina Lima Silva, Mary Bortoluzzi, Raquel Mantuaneli Scarel-Caminaga, Fabio Vianello

**Affiliations:** 1 Department of Comparative Biomedicine and Food Science, University of Padova, Viale dell’Università 16, 35020 Legnaro, PD, Italy; mary.bortoluzzi@phd.unipd.it (M.B.); fabio.vianello@unipd.it (F.V.); 2 Department of Morphology, Genetics, Orthodontics and Pediatric Dentistry, School of Dentistry at Araraquara, São Paulo State University—UNESP, Araraquara 14801-903, SP, Brazil; renata.cl.silva@unesp.br (R.C.L.S.); raquel.caminaga@unesp.br (R.M.S.-C.)

**Keywords:** bioactive peptides, anti-inflammatory, antimicrobial, periodontitis, diabetes, chronic diseases

## Abstract

Bioactive peptides have garnered increasing interest in recent years due to their potential applications in the medical field, for example, as promising adjuvant therapeutic agents to modulate the host immune response and counteract microbial dysbiosis in chronic pathologies. Primarily derived from protein hydrolysates of food waste, these components exhibit beneficial properties, such as anti-inflammatory, antimicrobial, antioxidant, and antidiabetic effects. This narrative review focuses on bioactive peptides with antimicrobial and anti-inflammatory properties, highlighting their mechanisms of action, sources, and therapeutic potential in the context of chronic conditions, particularly periodontal disease, especially when comorbidities are present (i.e., type 2 diabetes mellitus). The mechanisms of action and sources, as well as preclinical and clinical studies evaluating bioactive peptides efficacy, are discussed. Further research is warranted to establish their clinical viability and integration into conventional therapeutic strategies.

## 1. Introduction

**Figure 1:**
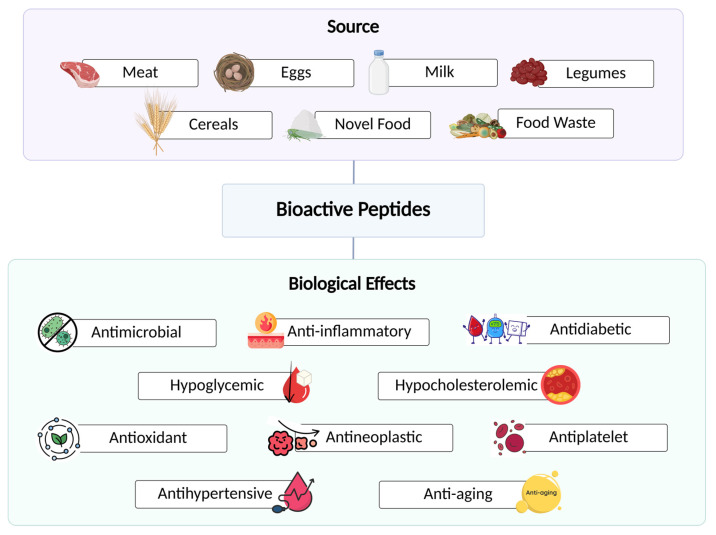
Sources of bioactive peptides and their associated health benefits.

Chronic diseases, such as cancer, diabetes, and cardiovascular conditions, are particularly challenging to manage due to their multifactorial etiology. As a result, nature-based therapies have become a significant focus of research in recent years. Among these, natural bioactive compounds, such as polyphenols and bioactive peptides (BPs), have increasingly attracted scientific interest. Bioactive peptides are fragments of proteins with relevant beneficial functions on human health and have a length ranging from 2 to 20 amino acid residues [1,2,3]. These molecules are isolated from both animal and plant sources, such as meat, eggs, milk, pulses, and cereals, but can also be obtained by food waste or novel foods, i.e., insects (Figure 1) [4,5,6].

More than 50 bioactivities of these peptides are described on the BIOPEP database [7]. A range of beneficial biological effects, such as antimicrobial, anti-inflammatory, antioxidant, antineoplastic, antihypertensive, antidiabetic, hypoglycemic, hypocholesterolemic, antiplatelet, and anti-aging effects have been reported for these components (Figure 1) [2,6,8].

These biological activities are of considerable clinical interest, and their therapeutic roles have been explored for various pathological conditions, including obesity [9], complications of periodontal diseases [10], and diabetes mellitus (DM) [11], in addition to their application in anti-cancer immunotherapy against glioblastoma, SARS-CoV-2, Parkinson’s disease [12], Alzheimer’s disease [13,14], and cardiovascular diseases [15,16].

Within this context, antimicrobial peptides (AMPs) offer a potential strategy for managing periodontal diseases by neutralizing bacterial toxins and limiting microbial overgrowth [17]. AMPs are small bioactive peptides acting as antimicrobial agents, which can be found in any organism, and, depending on their aminoacidic sequence and length, AMPs work as essential components of innate immunity [18]. These molecules, compared to antibiotics, have a lower tendency to develop resistant genes in pathogens when used as food additives or for prophylactic approaches [18]. For this reason, they have been studied as an interesting alternative to antibiotic administration.

Considering the inflammation of the gingival tissue that occurs in periodontitis, another potential therapeutic approach involves anti-inflammatory peptides (AIPs), which are molecules with immunomodulatory effects [19,20]. They play a role in cell differentiation, in the stimulation of wound healing, in microbial clearance and angiogenesis, and in preventing exacerbated immune responses, such as allergies and sepsis. Several AIPs showed positive effects when administered as anti-inflammatory medication. Some of them also have antioxidant properties, inhibiting reactive oxygen species (ROS), implicated in many diseases [8].

This narrative review aims to explore the therapeutic potential of bioactive peptides with antimicrobial and anti-inflammatory properties in the management of chronic diseases, particularly periodontal disease, and to assess their potential as supportive agents in combination with traditional therapies.

## 2. Methodology: Inclusion and Exclusion Criteria

In this narrative review, we considered peer-reviewed articles published in English between 2010 and 2025, while also including earlier seminal studies when they were particularly relevant. Eligible works comprised in vitro, in vivo, and clinical studies which investigated the antimicrobial and/or anti-inflammatory properties of bioactive peptides. We included research focusing on their therapeutic potential in periodontal disease, as well as in chronic conditions with relevance to periodontal pathogenesis, such as type 2 diabetes. Both original studies and reviews discussing mechanisms of action, sources, and clinical or preclinical evidence were analyzed.

Publications were excluded if they did not directly address bioactive peptides or lacked a clear focus on their antimicrobial or anti-inflammatory effects. Studies dealing exclusively with synthetic drugs, antibiotics, or non-peptide natural compounds (such as polyphenols or terpenes) were not considered. Articles written in languages other than English, as well as abstracts, conference proceedings, editorials, or commentaries without primary research data, were also excluded. Duplicate works and studies with insufficient methodological detail were omitted from the review.

## 3. Insights into Periodontal Disease: Diagnosis and Molecular Biomarkers

### 3.1. Diagnosis of Periodontal Diseases

Chronic local diseases, such as periodontal disease, are highly prevalent and significantly impact on the quality of life for millions of individuals worldwide [21].

The periodontium encompasses the supporting structures of the teeth, including the gingival and bone tissues, whose main function is to provide protection and stability. Gingiva is a protective structure, while the supporting tissues include the periodontal ligaments, cementum, and alveolar bone. When inflammation is confined to gingiva, the condition is referred to as gingivitis. In contrast, damage affecting deeper supporting tissues indicates progression to periodontitis [22,23]. Periodontitis is clinically manifested as clinical attachment loss, which is calculated by assessing the probing depth of periodontal pockets and the level of the gingiva. An increase in clinical attachment loss between periodontal evaluations indicates disease activity. Additional clinical indicators of disease activity include gingival bleeding and bleeding on probing (BOP), although their reliability can be limited in certain populations [21]. In particular, their diagnostic reliability is reduced in smokers, as tobacco-induced peripheral vasoconstriction may mask underlying inflammation [24].

**Figure 2:**
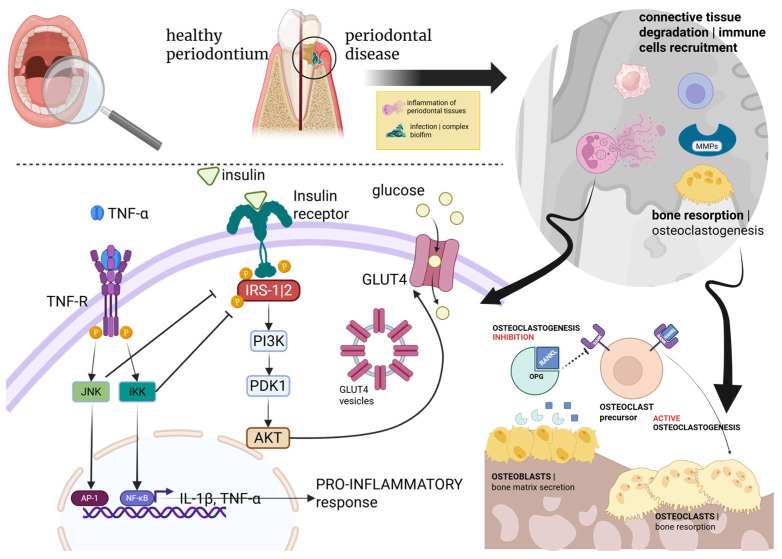
Schematic representation of the biological interrelationship between PD and DM.

Gingivitis and periodontitis are considered host-mediated immunoinflammatory disorders that occur in response to microbial dysbiosis of dental biofilm (Figure 2) [23,25]. The bacterial species primarily associated with the onset and progression of periodontitis include *Porphyromonas gingivalis*, *Tannerella forsythensis*, *Treponema denticola*, *Prevotella intermedia*, and *Aggregatibacter actinomycetemcomitans* [26]. This process upregulates several cytokines, mainly IL-1β (Interleukin-1-beta), IL-6, IL-17, IL-23, and TNF-α (Tumor Necrosis Factor-alpha) levels, leading to deeper infiltration of pro-inflammatory cells in periodontal tissues [26]. In other words, biofilm accumulation is usually necessary to trigger the pathological process, but the intensity of the tissue’s response to the microbial factor is dictated by the host’s immune system (Figure 2) [27].

If untreated, periodontal diseases lead to progressive destruction of both protective and supporting dental tissues, ultimately resulting in tooth loss. Indeed, periodontal diseases are the leading cause of tooth loss in the global adult population, affecting over 1 billion individuals in its severe form [28]. A meta-analysis estimated the overall prevalence of periodontal diseases as 61.6% in 17 countries around the world [29].

Periodontitis can be triggered by microorganism-derived dental biofilm and the consequent host’s immune response, involving recruitment of immune cells (e.g., neutrophils, lymphocytes, and macrophages), activation of lytic enzymes, such as matrix metalloproteinases (MMPs), and osteoclastogenesis via the RANK–RANKL–OPG axis (Figure 2) [30]. This signaling pathway plays a key role in bone metabolism, and it is regulated by the receptor activator of NF-κB (RANK), its ligand RANKL, and osteoprotegerin (OPG), a member of the TNF superfamily acting as a competitive inhibitor of RANKL and preventing its interaction with RANK [31]. Consequently, OPG activity leads to a reduction in RANK-mediated downstream effects, such as bone remodeling and osteoclast activation [30,31]. Periodontal disease can be further exacerbated in the presence of chronic comorbidities, such as diabetes. Indeed, the RANK–RANKL–OPG axis is subject to modulation by various hormones and cytokines, particularly TNF-α, which contributes to insulin resistance and a pro-inflammatory state [30,31]. Specifically, TNF-α binds to its receptor (TNF-R), activating two kinases, JNK and IKK, which in turn activate the transcription factors AP-1 and NF-κB (through degradation of its inhibitor, IκB), respectively [30,32]. These transcription factors translocate to the cell nucleus and induce the expression of pro-inflammatory cytokines, such as interleukin-1 beta (IL-1β) and TNF-α, creating a positive feedback loop sustaining inflammation. Moreover, insulin binds to the insulin receptor and activates IRS-1/2 (Insulin Receptor Substrates 1 and 2). It should be noted that, in addition to their chronic nature, a shared genetic architecture between periodontal disease and T2DM was demonstrated by many single nucleotide polymorphisms (SNPs), predisposing T2DM-affected people to periodontitis [33,34,35]. This connection was further supported by a comprehensive genome-wide analysis involving thousands of European individuals, demonstrating a genetic link between diseases and glycemic traits, influenced by biological pleiotropy. Pleiotropy, in this context, refers to the existence of common pathogenic pathways across different diseases, driven by shared genetic variants [36,37].

Taken together, these diagnostic and etiological insights provide a framework for exploring targeted therapeutic interventions, particularly those involving bioactive peptides.

### 3.2. Endogenous Peptides Acting as Markers of Periodontitis Pathogenesis

Beyond clinical classification and diagnosis, a deeper understanding of the biological mechanisms underlying periodontal disease is essential for developing targeted therapies. Among these mechanisms, endogenous peptides have emerged as promising molecular markers involved in both disease onset and progression.

Endogenous peptides play a unique and multifaceted role in the etiopathogenesis of periodontal disease. These peptides may participate in both host-derived (endogenous) and externally triggered (exogenous) mechanisms. They are implicated not only in initiating the immune response that drives the onset and progression of periodontitis, but also in modulating immunoinflammatory pathways linked to known risk factors for the disease (although the specific pathways involved remain unclear). Their presence is typically assessed in gingival crevicular fluid, saliva, or periodontal tissue samples. Several human bioactive markers relevant to periodontal diseases have been identified, including neutrophil peptides (HNP, alpha-defensins), beta-defensins (HBD), and LL-37 (cathelicidin) [38].

Saliva also represents a valuable, non-invasive source of bioactive antimicrobial peptides for the diagnosis of periodontal diseases. This fluid has been studied as an interesting and potential tool for the diagnosis of many diseases, with intraoral manifestations or not, such as dental caries, periodontal diseases, oral and breast cancer, and hepatitis [39].

While endogenous peptides serve as valuable diagnostic indicators, they also play a central role in guiding therapeutic strategies aimed at modulating immune responses and promoting tissue regeneration. This dual role underscores the importance of integrating biological insights into the design of effective periodontal therapies. For this purpose, the following section reviews the main therapeutic approaches used in periodontal diseases management, highlighting how such insights are being translated into practice.

### 3.3. Periodontal Therapy

Periodontal therapy can be broadly divided into nonsurgical (basic) and surgical phases. Nonsurgical therapy involves controlling supragingival biofilm and performing root scaling and planning (subgingival debridement) through multiple clinical sessions. Oral hygiene instructions are consistently reinforced throughout treatment. Depending on the treatment stage and the host’s response, adjunctive antibiotic therapy may be prescribed to enhance clinical outcomes. However, in some cases, initial therapy fails to achieve the desired results, particularly in reducing clinical attachment loss, making surgical intervention necessary. In these situations, surgical approaches, such as open flap debridement or the modified Widman flap, are employed to provide improved access to areas with residual biofilm and/or granulation tissue [40,41].

While periodontal basic therapy (root scaling and planning) is indispensable for all patients, including those with DM, numerous adjunctive therapies have been explored to enhance the efficacy of periodontal treatment by modulating the host’s immune response. A variety of studies have been conducted to assess the effects of different substances on periodontal cells and tissues. These include laser and photodynamic therapies [42,43], anti-inflammatory medications [44,45,46] ozone therapy [47,48,49], and natural agents, such as curcumin [50,51] and platelet derivatives [52], among others.

These therapeutic strategies provide a foundation for considering novel biological interventions, including the application of bioactive peptides, in managing periodontal inflammation and regeneration.

## 4. Bioactive Peptides in the Management of Chronic Diseases

### 4.1. Anti-Inflammatory Peptides (AIPs)

Inflammation is the body’s natural defense mechanism against infection or injury and can occur in both acute and chronic forms. Chronic inflammation, however, is particularly concerning as it is associated with a range of lifestyle-related diseases, including arthritis, type 2 diabetes, cardiovascular diseases, and inflammatory bowel disease [53]. Long-term reliance on medications to manage these conditions often leads to undesirable side effects, raising concerns about their prolonged use. As a result, there is increasing interest in food-derived AIPs as a potential alternative treatment [54].

The anti-inflammatory properties of bioactive peptides are primarily attributed to their hydrophobic and cationic nature, as noted in several studies [55,56,57]. Many of these peptides contain positively charged amino acids, such as arginine (Arg) and lysine (Lys), and often feature hydrophobic amino acids at the N-terminal end and polar amino acids at the C-terminal end. Specific amino acid sequences, including Val-His, Ile-Ala, Ile-Pro-Pro, Val-Pro-Pro, Ile-Arg-Trp, Ile-Gln-Trp, Phe-Leu-Val, Leu-Asp-Ala-Val-Asn-Arg, Val-Pro-Tyr, and Met-Met-Leu-Asp-Phe, have shown notable anti-inflammatory effects [55,56,57]. Moreover, the presence of methionine and cysteine has shown a decrease in the inflammation status in different trials [54].

**Figure 3:**
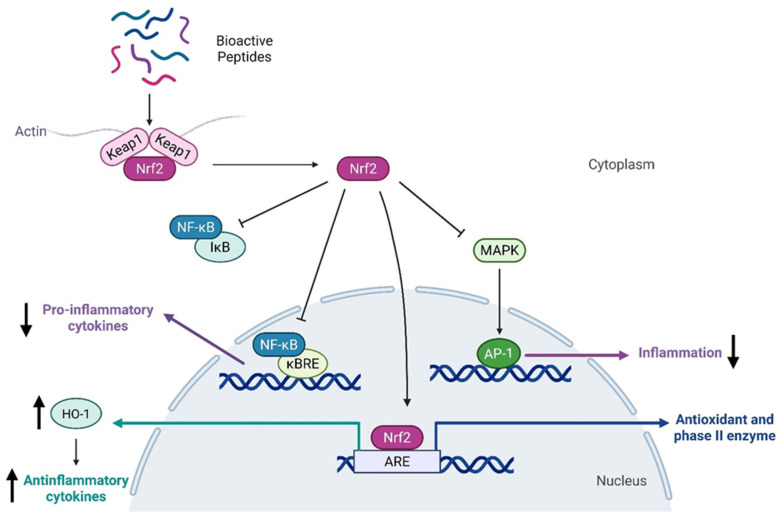
Schematic representation of the molecular mechanisms of bioactive peptides exhibiting antioxidant and anti-inflammatory properties.

These bioactive peptides exhibit anti-inflammatory effects by targeting critical pathways such as NF-κB (Nuclear Factor kappa-light-chain-enhancer of activated B cells) and MAPK (Mitogen-Activated Protein Kinase), both of which play fundamental roles in sustaining chronic inflammation [54,55,58]. For example, a peptide derived from casein, containing the sequence Gln-Glu-Pro-Val, has been found to inhibit nitric oxide production, while increasing the secretion of anti-inflammatory cytokines IL-4 and IL-10 [59]. Moreover, some peptides derived from milk products have shown particular benefits in managing inflammation status due to their antioxidative and anti-inflammatory properties, as confirmed by multiple studies [60,61]. This aspect was demonstrated to be correlated with the activation of the pathway regulating redox homeostasis, which is modulated by Kelch-like ECH-associated protein 1 (Keap1) and the Nuclear factor erythroid 2-related factor 2 (Nrf2), and the action of the latter on Nrf2/ARE/HO-1 axis, which is involved in the inhibition of NF-κB pathway [62]. The molecular mechanisms activated by bioactive peptides are reported in Figure 3.

Peptides from different sources, such as milk, sunflower, garlic, and meat, have been revealed to be able to activate Keap1/Nrf2 axis, showing antioxidant properties and at the same time anti-inflammatory abilities due to the consequent inhibition of the pro-inflammatory pathway mediated by NF-κB, with a consequent decrease in cytokine release [60,63,64,65].

### 4.2. Antimicrobial Peptides (AMPs)

In addition to their anti-inflammatory potential, bioactive peptides also exhibit potent antimicrobial properties that are of growing interest, especially in the context of increasing antibiotic resistance. For this purpose, this section explores antimicrobial peptides (AMPs), highlighting their mechanisms of action and relevance in the management of microbial infections. Indeed, given the alarming rise of resistance to antimicrobial agents, research is increasingly focused on identifying new molecules with antimicrobial properties and a lower likelihood of developing resistance. In this context, AMPs emerge as a promising solution [66].

AMPs are bioactive peptides containing up to around 50 amino acid residues and are found across a wide range of sources, representing a primary barrier against microbial invasions [67]. These peptides include both hydrophobic and hydrophilic amino acids at their N- and C-terminal, which are recognized as structural motifs allowing their interaction with microbes [68]. AMPs have broad-spectrum antimicrobial activity, in addition to plenty of other biological functions, such as enhancing animal growth, regulating animal morphology and its microbiota, and optimizing animal disease resistance [69]. Their significance lies in the fact that, unlike traditional antibiotics, microbial resistance to AMPs develops much more slowly [70]. Their natural presence, dating back millions of years, highlights their effectiveness in combating infections [71].

**Figure 4:**
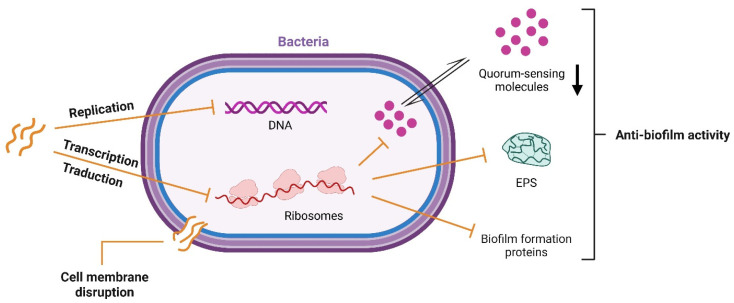
Schematic representation of targets for antimicrobial peptides.

AMPs are distinguished by their structural and functional diversity, with the ability to vary in sequence, structure, and mechanism of action on microbial targets [72]. These peptides exert their antimicrobial activity by different mechanisms, including interference with intracellular macromolecules, disruption of bacterial membrane, interaction with microbial signaling pathways, and host microbiota modulation (Figure 4).

Most AMPs are cationic and have amphipathic conformation, enabling their electrostatic interaction with the negatively charged regions on the bacterial membrane. Notably, hydrophobic amino acid residues, such as valine, leucine, isoleucine, methionine, phenylalanine, tyrosine, and tryptophan, can affect cell membrane permeability due to their hydrophobic feature [18]. Indeed, these AMPs can be easily integrated into the lipid bilayer, forming transmembrane pores damaging bacterial membrane integrity (Figure 4) [73]. It is worth noting that AMPs are also produced by Gram-positive and Gram-negative bacteria via ribosomal synthesis to inhibit the growth of competing bacterial species. These peptides are known as bacteriocins, and their study is of growing interest due to their potential as therapeutic agents against antibiotic resistance [74]. Moreover, some bioactive peptides with inhibitory activities during the production of extracellular polymeric substances (EPS) were studied to prevent biofilm formation [75]. In particular, Luo et al. (2021) described that AMPs can affect the quorum-sensing system, blocking the bacteria’s mechanism of communication necessary for biofilm formation [76] (Figure 4). Furthermore, peptides can downregulate the expression of binding protein transport for genes employed in biofilm formation, and also interfere with the synthesis of enzymes necessary for biofilm structure [76].

Some antimicrobial peptides (AMPs) induce cell lysis through electrostatic interactions with the microbial membrane, disrupting membrane permeability and inhibiting protein, RNA, and DNA synthesis. Moreover, some AMPs can penetrate the cell and interfere directly with replication and transcription by targeting DNA or RNA (Figure 4) [77,78].

For instance, peptides derived from caseins, in particular α-1, have been shown to act on many varieties of Gram-positive bacteria, including *Staphylococcus aureus* [79].

The majority of peptides (86%, Apr 2025) present in the Antimicrobial Peptide Database (APD) are antibacterial [80]. A specific example of AMP activity comes from a study by Segura-Campos et al. (2013), which demonstrated that the hydrolyzed peptide from chia flour (*Salvia hispanica* L.) inhibited the growth of both Gram-negative bacteria (such as *Escherichia coli*, *Salmonella typhi*, and *Shigella flexneri*) and Gram-positive bacteria (such as *Klebsiella pneumoniae*, *Staphylococcus aureus*, *Bacillus subtilis*, and *Streptococcus agalactiae*) [81]. Another key study conducted by Abadía-García et al. (2013) examined AMP release in a food matrix, demonstrating that probiotic microorganisms added to ricotta cheese under gastrointestinal-like conditions inhibited the foodborne pathogen *Listeria monocytogenes* [82]. They observed that the population of *L. monocytogenes* was reduced by a full logarithm at the end of the 20-day storage period [82]. Innovative methods are currently applied for AMP detection, such as machine learning and a language-based deep generative framework [83,84]. Despite their potential as an alternative to antibiotics, there are challenges associated with the delivery and stability of antimicrobial peptides that have to be assessed to finally propose them as drugs [85].

Taken together, the immunomodulatory and antimicrobial properties of these peptides provide a compelling rationale for their application in complex diseases, such as periodontitis. In this context, both antimicrobial peptides and anti-inflammatory peptides have been investigated as adjunctive treatments to support periodontal therapy.

### 4.3. AMP and AIP Peptides in Periodontal Diseases and DM

The interplay between microbial dysbiosis and host inflammatory response is central to periodontal disease progression. Therefore, bioactive peptides targeting both processes represent a novel therapeutic avenue. Some reviews address the potential and importance of BPs, from various sources, in the general dental field [73,86,87,88,89].

Antimicrobial BPs can even be endogenously produced, appearing as important markers of periodontal diseases. Their activity, such as in the case of defensins, is mainly immunomodulatory [90]. However, in most cases, the host’s response is not enough to control periodontitis inflammation, and conventional periodontal therapy is required to control infection.

It is widely known that individuals with T2DM, particularly those with elevated glycated hemoglobin levels, exhibit worsened and more severe periodontal disease, which can be difficult to manage. Even after periodontal treatments, these patients may present sites with progressive clinical attachment loss [91]. In such cases, adjunctive therapies could be beneficial by promoting antimicrobial activity against periodontopathogens and reducing the exacerbated inflammatory response, without the need for systemic antibiotics and their associated side effects [89].

This section explores the applications of AIPs and AMPs in preclinical and clinical studies to improve outcomes in periodontal disease, particularly in patients with comorbid conditions, such as diabetes.

#### 4.3.1. In Vitro Studies on the Effects of BPs on Periodontal Diseases

Due to the infectious nature of periodontal diseases, adjunctive approaches helping to handle the action of periodontopathogens have been widely studied in recent years [92,93,94]. Several in vitro studies have explored the potential of bioactive peptides in modulating inflammation and in promoting the regeneration of periodontal cells. Mineo et al. (2021) explored the in vitro (gingival cells) antioxidant and anti-inflammatory activity of protein fractions from rice bran associated with the action of sulforaphane (SFN), a potent antioxidant molecule, confirming the anti-inflammatory properties of rice-derived peptides [95]. They concluded that peptides from rice bran enhanced SFN-induced antioxidant responses in epithelial cells from the gingiva by ERK-Nrf2-ARE signaling [95]. In another study, Tamura et al. (2019) investigated the activity of peptides from rice endosperm on the gene expression of molecules related to inflammation and osteoclastogenesis processes [96]. They found that peptides REP9 and REP11 from rice could reduce the transcription of proinflammatory molecules, such as IL-6, Nlrp3, and TNF, and osteoclast differentiation markers (Nfatc1, RANK). The peptide REP9 was also able to upregulate Bcl6 expression, a marker connected to the suppression of osteoclast differentiation and function [96].

Synthetic peptides have also demonstrated promising effects. One study involving synthetic ADP-5 (Amelogenin-derived peptide) enhanced proliferation, migration, and regenerative marker expression in gingival fibroblasts, periodontal ligament cells, osteoblasts, and cementoblasts, indicating that this peptide may support periodontal tissue regeneration and could be applied in biomaterial-based therapies for periodontal repair [97]. Another synthetic component, the peptide CH02, was reported to promote osteogenic differentiation of human periodontal ligament cells by increasing the expression of osteogenic markers, highlighting its potential as a bioactive agent for stimulating bone regeneration in periodontal therapy [98].

#### 4.3.2. In Vivo Studies on the Effects of BPs on Periodontal Diseases

Animal models have provided further evidence of the therapeutic potential of bioactive peptides. Tamura et al. (2019), in the animal model section of their work, indicated that peptides REP9 and REP11 from rice were able to inhibit bone loss, reducing TRAP-positive osteoclasts of the alveolar bone perimeter in their histological analysis [96]. Another interesting contribution to this field was proposed by Yuexiang Li et al. (2023), who investigated a polypeptide composed of an anti-inflammatory peptide (C15) and an antimicrobial peptide (LL37) in an animal model [99]. Their study revealed that the LL37-C15 polypeptide structure can prevent the binding and the growth of pathogenic bacteria by inhibiting the formation of biofilm, which causes oral dysbiosis, and decreasing factors leading to periodontitis. The anti-inflammatory activity was explained by the modulation of the production of pro- and anti-inflammatory cytokines. The tested animal model of periodontitis treated with LL37-C15 demonstrated promising therapeutic effects, suggesting that polypeptides could represent a new promising therapeutic approach [99].

Bomidin, a new recombinant antimicrobial peptide, was investigated for its effects on the inflammation of periodontal ligament stem cells (PDLSCs) stimulated by TNF-α. The study demonstrated that bomidin effectively suppressed inflammation by down-regulating the MAPK and NF-κB signaling pathways. Additionally, bomidin inhibited ferroptosis and activated the Keap1/Nrf2 pathway, suggesting its potential as a therapeutic agent in periodontal tissue inflammation [100]. Another recent study evaluated the clinical efficacy of CPNE7-DP in dogs with naturally occurring periodontitis. Topical application of CPNE7-DP, in conjunction with nonsurgical periodontal treatments, resulted in significant reductions in gingival inflammation, probing pocket depth, clinical attachment level, and alveolar bone loss. These findings suggest that CPNE7-DP can be proposed as an effective adjunctive therapeutic agent [101].

#### 4.3.3. Clinical Trials on the Effects of BPs on Periodontal Diseases

Regarding the clinical application of AMPs, Zhang et al. (2024) recruited 86 periodontitis patients and divided them into two equal and randomized groups to test a biological antibacterial polypeptide gel at the periodontium, together with periodontal basic therapy [102]. Individuals who received the AMP gel inside their periodontal pockets showed a significant decrease in the proinflammatory TNF-α and IL-6 levels in the gingival crevicular fluid, and an important improvement in periodontal parameters. Moreover, the levels of omentin-1, a cytokine with anti-inflammatory properties, were elevated in these groups compared to the control, confirming the beneficial effect of this application [102].

A study by Xiang et al. [103] investigated the efficacy of AMPs as an adjunct to scaling and root planning (SRP) in treating Stage III Grade B periodontitis. In this randomized clinical trial, 51 patients were divided into groups: SRP alone, SRP with minocycline hydrochloride (control), and SRP with AMPs (test). After 90 days, the AMP-treated group exhibited a significantly greater reduction in periodontal probing depth and an increase in clinical attachment compared to both SRP- and minocycline-treated groups, in addition to a notable decrease in periodontal pathogens and an increase in beneficial subgingival microbiota. Another study involving AMPs [104] evaluated the effectiveness of an oral spray containing the antimicrobial peptide P-113 in reducing oral bacteria and dental plaque formation. This trial involved 28 participants, who used either P-113-containing oral spray or a placebo over a 4-week period. Results indicated a significant reduction in the numbers of *Streptococcus* and *Porphyromonas* species in the experimental group, along with decreased dental plaque weight, dental plaque index, and gingival index. Additionally, 91.8% of participants reported satisfaction with the use of the proposed product. In a study involving another potential approach, which is now applied in periodontal surgeries, Jalali et al. investigated the effect of BPs purified from the algae *Spirulina platensis* on wound healing after periodontal flap surgery [105]. This study included 20 patients with periodontitis, who received either a gel containing *Spirulina* peptides or a placebo. The peptide-treated group exhibited significant reduction in plaque and bleeding indices at 4 and 8 weeks postoperatively, decreased gingival redness during the first week, and reduced pain, indicating improved healing and reduced inflammation. Additionally, no adverse effects or allergic reactions were observed, suggesting that the *Spirulina* peptide gel is safe and well-tolerated.

#### 4.3.4. Effects of Bioactive Peptides on Risk Factors for Periodontal Diseases

Other food-derived peptides may help the treatment of periodontal disease in patients affected by concomitant T2DM. As an example, dipeptidyl peptidase IV (DPP-IV) and α-glucosidase are enzymes with relevance to the pathogenesis of T2DM, and their inhibition could be a strategy to manage the condition. There are a variety of peptides with this activity, such as glipzide XL, glyburide, and glimepiride [106]. DPP-IV is a serine exopeptidase expressed on the surface of many cells, like transmembrane glycoprotein, or in soluble form in some body fluids, i.e., blood plasma. This enzyme cleaves X-proline or X-alanine dipeptides from the N-terminus of polypeptides, and is present in different human organs, such as the intestine, lungs, and kidneys [15]. It has been shown that some milk-derived peptides have a positive influence on the control of T2DM in several ways: decreasing appetite, regulating plasma glucose levels, and inhibiting glucose synthesis [106]. Drummond et al. (2018) and Li et al. (2023) demonstrated that different milk-derived bioactive peptides can act on glycemic management, modulating glucose uptake and metabolism [107,108]. Moreover, Santos-Hernandez et al. (2023) showed that milk- and egg-derived peptides enhanced the release of cholecystokinin (CCK) and glucagon-like peptide-1 (GLP-1), gastrointestinal hormones regulating satiety signaling in enteroendocrine cells [109]. On the other hand, specific signaling pathways can also be involved in the regulation of antidiabetic activity. For example, according to De Campos Zani et al. (2022) the tripeptide IRW (isoleucine–arginine–tryptophan) improved glucose tolerance and insulin responsiveness by activating the Insulin/AKT/GLUT4 signaling cascade in skeletal muscle [110]. The same effects were observed by Sharkey et al. (2020) using fish protein hydrolysate-derived bioactive peptides [111]. Several reviews have addressed this topic, describing the activity of bioactive peptides derived from vegetal products, such as legumes [112], amaranth, quinoa [113], and soybean [114], in both endogenous and exogenous pathways (Table 1).

Thus, by integrating both antimicrobial and anti-inflammatory actions, bioactive peptides offer a multifaceted approach to disease management, with particular relevance to chronic inflammatory disorders, such as periodontal disease, even under comorbidities.

**Figure 5:**
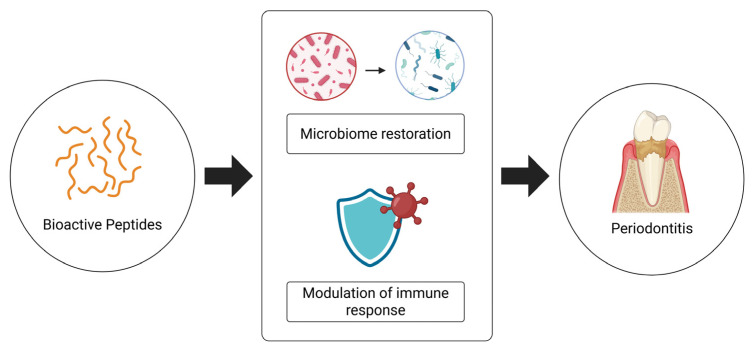
Impact of bioactive peptides in the treatment of periodontitis and human health.

Overall, these findings underscore the therapeutic versatility of bioactive peptides and their potential to serve as adjuncts in the treatment of complex, inflammation-driven diseases, such as periodontitis (Figure 5), especially in patients with comorbidities (type 2 diabetes mellitus). Further research is needed to translate these molecular insights into clinically viable interventions encompassing a large number of subjects.

## 5. Future Directions for Bioactive Peptides in the Management of Periodontal Disease and DM

Despite the accumulation of evidence, research on bioactive peptides (BPs) in the context of periodontal diseases and diabetes mellitus (DM) remains in its early stages. Mechanical biofilm removal through periodontal basic therapy continues to represent the cornerstone of care and is still irreplaceable [115,116,117]. Nonetheless, BPs exhibit antimicrobial, anti-inflammatory, and metabolic properties, making them attractive as adjunctive therapeutic agents, particularly in patients with comorbidities, such as type 2 DM. Current barriers to clinical translation include limited stability, suboptimal systemic absorption, heterogeneity in reported outcomes, insufficient data on dose–response relationships, long-term safety, and cost-effectiveness. Additional concerns are related to potential toxicity, drug interactions, and to the absence of standardized delivery systems capable of ensuring peptide stability and targeted bioavailability.

Future research should adopt a systematic trajectory, progressing from in vitro characterization to in vivo validation and, ultimately, to large-scale applications and rigorously designed clinical trials assessing both periodontal and systemic endpoints, including glycemic control. The elucidation of the molecular mechanisms by which BPs modulate host immunity and microbial dysbiosis will be critical for establishing mechanistic plausibility. Parallel efforts should focus on the overcoming of pharmacokinetic limitations using innovative delivery approaches, such as hydrogels, nanoparticles, and mucoadhesive formulations [118]. Moreover, the evaluation of synergistic effects with established modalities, such as scaling and root planning, antibiotics, and antidiabetic therapies, may enhance therapeutic efficacy in complex clinical scenarios.

The integration of precision medicine strategies incorporating genetic susceptibility, microbiome profiles, and systemic conditions could further refine patient stratification and optimize outcomes. Finally, attention to regulatory frameworks and scalable biotechnological production will be indispensable to facilitate clinical translation. Collectively, addressing these research priorities will be essential to establish BPs as credible, innovative, and sustainable therapeutic tools for the management of periodontal disease and its systemic associations, with the potential to significantly improve patient prognosis and quality of life.

## 6. Conclusions

Bioactive peptides endowed with antimicrobial and anti-inflammatory properties represent a promising adjunctive strategy for managing periodontal disease, particularly in the presence of comorbidities such as T2DM. Their dual ability, the modulation of host immune responses and the contrast of microbial dysbiosis, highlights their potential to improve clinical outcomes. It is important to emphasize that their role should be considered as adjunctive therapy in addition to mechanical biofilm removal. However, further research is needed to clarify mechanisms of action, to assess safety, and to optimize delivery systems. Despite these challenges, bioactive peptides offer an innovative avenue for future therapies in complex, inflammation-driven diseases.

## Figures and Tables

**Table 1:** Key bioactive peptides discussed in this review, their sources, and potential therapeutic effects on periodontal diseases.

Bioactive Peptide	Source	Potential Therapeutic Effect on Periodontal Diseases/T2DM	Ref.
K-8-K, S-10-S	Milk	Antioxidant and anti-inflammatory	[60]
SPI (sunflower protein isolate)	Sunflower	Anti-inflammatory	[63]
Garlic bioactive proteins	Garlic	Anti-inflammatory	[64]
Chia hydrolysates	Chia	Antimicrobial activity	[81]
REP9, REP11	Rice	Anti-inflammatory and inhibition of osteoclastogenesis	[95,96]
LL37-C15	Endogenous (human catelicidin)	Anti-inflammatory	[99]
Milk-derived hydrolysates	Milk (casein hydrolysates and rubing cheese)	Antidiabetic–glycemic management	[106,107,108]

## Data Availability

Not applicable.

## Abbreviations

BPs = Bioactive Peptides; AMPs = Antimicrobial Peptides; AIPs = Anti-Inflammatory Peptides; ROS = Reactive Oxygen Species; T2DM = Type 2 Diabetes Mellitus; BOP = Bleeding On Probing; LPSs = Lypopolissacharides; TNF-α = Tumor Necrosis Factor-alpha; TNF-R = Tumor Necrosis Factor Receptor; JNK = c-Jun N-terminal Kinase; IKK = IκB Kinase; NF-κB =Nuclear Factor kappa-light-chain-enhancer of activated B cells; AP-1 = Activator Protein-1; IRS-1/IRS-2 = Insulin Receptor Substrate 1 and 2; PI3K = Phosphoinositide 3-Kinase; PDK-1 = 3-Phosphoinositide-dependent Protein Kinase-1; AKT = Protein Kinase B; GLUT4 = Glucose Transporter Type 4; IL-1β = Interleukin-1 beta; MMPs = Matrix MetalloProteinases; RANK = Receptor Activator of Nuclear Factor κB; RANKL = Receptor Activator of Nuclear Factor κB Ligand; OPG = Osteoprotegerin; Keap1 = Kelch-like ECH-associated protein 1; Nrf2 = Nuclear factor erythroid 2-related factor 2; IκB: Inhibitor of nuclear factor kappa B; ARE: Antioxidant Response Element; κBRE: κB Response Element; HO-1: Heme Oxygenase-1, MAPK: Mitogen-Activated Protein Kinase; EPSs = Extracellular Polymeric Substances; APD = Antimicrobial Peptide Database.

## References

[1] Zaky A.A., Simal-Gandara J., Eun J.B., Shim J.H., Abd El-Aty A.M. (2022). Bioactivities, Applications, Safety, and Health Benefits of Bioactive Peptides From Food and By-Products: A Review. Front. Nutr..

[2] Du Z., Li Y. (2022). Review and Perspective on Bioactive Peptides: A Roadmap for Research, Development, and Future Opportunities. J. Agric. Food Res..

[3] Sánchez A. (2017). Alfredo Vázquez Bioactive Peptides: A Review. Food Qual. Safety.

[4] Manzoor M., Singh J., Gani A. (2022). Exploration of Bioactive Peptides from Various Origin as Promising Nutraceutical Treasures: In Vitro, In Silico and In Vivo Studies. Food Chem..

[5] Akbarian M., Khani A., Eghbalpour S., Uversky V.N. (2022). Bioactive Peptides: Synthesis, Sources, Applications, and Proposed Mechanisms of Action. Int. J. Mol. Sci..

[6] Colombo R., Pellicorio V., Barberis M., Frosi I., Papetti A. (2024). Recent Advances in the Valorization of Seed Wastes as Source of Bioactive Peptides with Multifunctional Properties. Trends Food Sci. Technol..

[7] Minkiewicz P., Iwaniak A., Darewicz M. (2022). BIOPEP-UWM Virtual—A Novel Database of Food-Derived Peptides with In Silico-Predicted Biological Activity. Appl. Sci..

[8] Dadar M., Shahali Y., Chakraborty S., Prasad M., Tahoori F., Tiwari R., Dhama K. (2019). Antiinflammatory Peptides: Current Knowledge and Promising Prospects. Inflamm. Res..

[9] de Medeiros A.F., de Queiroz J.L.C., Maciel B.L.L., de Araújo Morais A.H. (2022). Hydrolyzed Proteins and Vegetable Peptides: Anti-Inflammatory Mechanisms in Obesity and Potential Therapeutic Targets. Nutrients.

[10] Silva J.D., Leal E.C., Carvalho E. (2021). Bioactive Antimicrobial Peptides as Therapeutic Agents for Infected Diabetic Foot Ulcers. Biomolecules.

[11] Luong A.D., Buzid A., Luong J.H.T. (2022). Important Roles and Potential Uses of Natural and Synthetic Antimicrobial Peptides (AMPs) in Oral Diseases: Cavity, Periodontal Disease, and Thrush. J. Funct. Biomater..

[12] Martini S., Tagliazucchi D. (2023). Bioactive Peptides in Human Health and Disease. Int. J. Mol. Sci..

[13] Zhao L., Li D., Qi X., Guan K., Chen H., Wang R., Ma Y. (2022). Potential of Food-Derived Bioactive Peptides in Alleviation and Prevention of Alzheimer’s Disease. Food Funct..

[14] Wei M., Wu T., Chen N. (2024). Bridging Neurotrophic Factors and Bioactive Peptides to Alzheimer’s Disease. Ageing Res. Rev..

[15] Cruz-Chamorro I., Santos-Sánchez G., Bollati C., Bartolomei M., Capriotti A.L., Cerrato A., Laganà A., Pedroche J., Millán F., del Carmen Millán-Linares M. (2023). Chemical and Biological Characterization of the DPP-IV Inhibitory Activity Exerted by Lupin (*Lupinus angustifolius*) Peptides: From the Bench to the Bedside Investigation. Food Chem..

[16] Ganguly A., Sharma K., Majumder K. (2019). Food-Derived Bioactive Peptides and Their Role in Ameliorating Hypertension and Associated Cardiovascular Diseases. Adv. Food Nutr. Res..

[17] Gorr S.U., Abdolhosseini M. (2011). Antimicrobial Peptides and Periodontal Disease. J. Clin. Periodontol..

[18] Aslam M.Z., Firdos S., Li Z., Wang X., Liu Y., Qin X., Yang S., Ma Y., Xia X., Zhang B. (2022). Detecting the Mechanism of Action of Antimicrobial Peptides by Using Microscopic Detection Techniques. Foods.

[19] Mark Bartold P., Van Dyke T.E. (2013). Periodontitis: A Host-Mediated Disruption of Microbial Homeostasis. Unlearning Learned Concepts. Periodontol 2000.

[20] Budala D.G., Martu M.-A., Maftei G.-A., Diaconu-Popa D.A., Danila V., Luchian I. (2023). The Role of Natural Compounds in Optimizing Contemporary Dental Treatment—Current Status and Future Trends. J. Funct. Biomater..

[21] Baeza M., Morales A., Cisterna C., Cavalla F., Jara G., Isamitt Y., Pino P., Gamonal J. (2020). Effect of Periodontal Treatment in Patients with Periodontitis and Diabetes: Systematic Review and Meta-Analysis. J. Appl. Oral Sci..

[22] Chapple I.L.C., Mealey B.L., Van Dyke T.E., Bartold P.M., Dommisch H., Eickholz P., Geisinger M.L., Genco R.J., Glogauer M., Goldstein M. (2018). Periodontal Health and Gingival Diseases and Conditions on an Intact and a Reduced Periodontium: Consensus Report of Workgroup 1 of the 2017 World Workshop on the Classification of Periodontal and Peri-Implant Diseases and Conditions. J. Periodontol..

[23] Kinane D.F., Stathopoulou P.G., Papapanou P.N. (2017). Periodontal Diseases. Nat. Rev. Dis. Primers.

[24] Heitz-Mayfield L.J.A. (2024). Conventional Diagnostic Criteria for Periodontal Diseases (Plaque-induced Gingivitis and Periodontitis). Periodontol 2000.

[25] Abusleme L., Hoare A., Hong B., Diaz P.I. (2021). Microbial Signatures of Health, Gingivitis, and Periodontitis. Periodontol 2000.

[26] Usui M., Onizuka S., Sato T., Kokabu S., Ariyoshi W., Nakashima K. (2021). Mechanism of Alveolar Bone Destruction in Periodontitis—Periodontal Bacteria and Inflammation. Jpn. Dent. Sci. Rev..

[27] Belibasakis G.N., Belstrøm D., Eick S., Gursoy U.K., Johansson A., Könönen E. (2023). Periodontal Microbiology and Microbial Etiology of Periodontal Diseases: Historical Concepts and Contemporary Perspectives. Periodontol 2000.

[28] Nascimento G.G., Alves-Costa S., Romandini M. (2024). Burden of Severe Periodontitis and Edentulism in 2021, with Projections up to 2050: The Global Burden of Disease 2021 Study. J. Periodontal. Res..

[29] Trindade D., Carvalho R., Machado V., Chambrone L., Mendes J.J., Botelho J. (2023). Prevalence of Periodontitis in Dentate People between 2011 and 2020: A Systematic Review and Meta-Analysis of Epidemiological Studies. J. Clin. Periodontol..

[30] Valverde A., George A., Nares S., Naqvi A.R. (2025). Emerging Therapeutic Strategies Targeting Bone Signaling Pathways in Periodontitis. J. Periodontal Res..

[31] Monti F., Perazza F., Leoni L., Stefanini B., Ferri S., Tovoli F., Zavatta G., Piscaglia F., Petroni M.L., Ravaioli F. (2024). RANK–RANKL–OPG Axis in MASLD: Current Evidence Linking Bone and Liver Diseases and Future Perspectives. Int. J. Mol. Sci..

[32] Ru L., Pan B., Zheng J. (2023). Signalling Pathways in the Osteogenic Differentiation of Periodontal Ligament Stem Cells. Open Life Sci..

[33] Cirelli T., Nicchio I.G., Bussaneli D.G., Silva B.R., Nepomuceno R., Orrico S.R.P., Cirelli J.A., Theodoro L.H., Barros S.P., Scarel-Caminaga R.M. (2023). Evidence Linking PPARG Genetic Variants with Periodontitis and Type 2 Diabetes Mellitus in a Brazilian Population. Int. J. Mol. Sci..

[34] Cirelli T., Nepomuceno R., Rios A.C.S., Orrico S.R.P., Cirelli J.A., Theodoro L.H., Barros S.P., Scarel-Caminaga R.M. (2020). Genetic Polymorphisms in the Interleukins IL1B, IL4, and IL6 Are Associated with Concomitant Periodontitis and Type 2 Diabetes Mellitus in Brazilian Patients. J. Periodontal Res..

[35] Cirelli T., Nepomuceno R., Goveia J.M., Orrico S.R.P., Cirelli J.A., Theodoro L.H., Barros S.P., Scarel-Caminaga R.M. (2021). Association of Type 2 Diabetes Mellitus and Periodontal Disease Susceptibility with Genome-Wide Association–Identified Risk Variants in a Southeastern Brazilian Population. Clin. Oral. Investig..

[36] Wu K.C.H., Liu L., Xu A., Chan Y.H., Cheung B.M.Y. (2024). Shared Genetic Architecture between Periodontal Disease and Type 2 Diabetes: A Large Scale Genome-Wide Cross-Trait Analysis. Endocrine.

[37] Loos B.G., Van Dyke T.E. (2020). The Role of Inflammation and Genetics in Periodontal Disease. Periodontol 2000.

[38] Li S., Schmalz G., Schmidt J., Krause F., Haak R., Ziebolz D. (2018). Antimicrobial Peptides as a Possible Interlink between Periodontal Diseases and Its Risk Factors: A Systematic Review. J. Periodontal. Res..

[39] Güncü G.N., Yilmaz D., Könönen E., Gürsoy U.K. (2015). Salivary Antimicrobial Peptides in Early Detection of Periodontitis. Front. Cell Infect. Microbiol..

[40] Schulz S., Stein J.M., Schumacher A., Kupietz D., Yekta-Michael S.S., Schittenhelm F., Conrads G., Schaller H.-G., Reichert S. (2022). Nonsurgical Periodontal Treatment Options and Their Impact on Subgingival Microbiota. J. Clin. Med..

[41] Graziani F., Karapetsa D., Alonso B., Herrera D. (2017). Nonsurgical and Surgical Treatment of Periodontitis: How Many Options for One Disease?. Periodontol 2000.

[42] da Silva-Junior P.G.B., Abreu L.G., Costa F.O., Cota L.O.M., Esteves-Lima R.P. (2023). The Effect of Antimicrobial Photodynamic Therapy Adjunct to Non-Surgical Periodontal Therapy on the Treatment of Periodontitis in Individuals with Type 2 Diabetes Mellitus: A Systematic Review and Meta-Analysis. Photodiagnosis Photodyn. Ther..

[43] Salvi G.E., Stähli A., Schmidt J.C., Ramseier C.A., Sculean A., Walter C. (2020). Adjunctive Laser or Antimicrobial Photodynamic Therapy to Non-surgical Mechanical Instrumentation in Patients with Untreated Periodontitis: A Systematic Review and Meta-analysis. J. Clin. Periodontol..

[44] Li N., Xie L., Wu Y., Wu Y., Liu Y., Gao Y., Yang J., Zhang X., Jiang L. (2022). Dexamethasone-Loaded Zeolitic Imidazolate Frameworks Nanocomposite Hydrogel with Antibacterial and Anti-Inflammatory Effects for Periodontitis Treatment. Mater. Today Bio.

[45] Silva R.C.L., Sasso-Cerri E., Cerri P.S. (2022). Diacerein-induced Interleukin-1β Deficiency Reduces the Inflammatory Infiltrate and Immunoexpression of Matrix Metalloproteinase-8 in Periodontitis in Rat Molars. J. Periodontol..

[46] Jain P., Mirza M.A., Talegaonkar S., Nandy S., Dudeja M., Sharma N., Anwer M.K., Alshahrani S.M., Iqbal Z. (2020). Design and in Vitro/in Vivo Evaluations of a Multiple-Drug-Containing Gingiva Disc for Periodontotherapy. RSC Adv..

[47] Ramirez-Peña A.M., Sánchez-Pérez A., Campos-Aranda M., Hidalgo-Tallón F.J. (2022). Ozone in Patients with Periodontitis: A Clinical and Microbiological Study. J. Clin. Med..

[48] D′Ambrosio F., Caggiano M., Acerra A., Pisano M., Giordano F. (2023). Is Ozone a Valid Adjuvant Therapy for Periodontitis and Peri-Implantitis? A Systematic Review. J. Pers. Med..

[49] Uraz A., Karaduman B., Isler S.Ç., Gönen S., Çetiner D. (2019). Ozone Application as Adjunctive Therapy in Chronic Periodontitis: Clinical, Microbiological and Biochemical Aspects. J. Dent. Sci..

[50] Terby S., Shereef M., Ramanarayanan V., Balakrishnan B. (2021). The Effect of Curcumin as an Adjunct in the Treatment of Chronic Periodontitis: A Systematic Review and Meta-Analysis. Saudi Dent. J..

[51] Mohammad C.A., Ali K.M., Sha A.M., Gul S.S. (2022). Antioxidant Effects of Curcumin Gel in Experimental Induced Diabetes and Periodontitis in Rats. Biomed. Res. Int..

[52] Hurjui I., Delianu C., Liliana H.L., Raluca J., Mihaela M., Carina B., Oana A.A., Alexandra M.M., Irina G. (2020). Platelet Derivatives with Dental Medicine Applications. Rom. J. Oral. Rehabil..

[53] Serhan C.N., Savill J. (2005). Resolution of Inflammation: The Beginning Programs the End. Nat. Immunol..

[54] Biji C.A., Balde A., Nazeer R.A. (2024). Anti-Inflammatory Peptide Therapeutics and the Role of Sulphur Containing Amino Acids (Cysteine and Methionine) in Inflammation Suppression: A Review. Inflamm. Res..

[55] Guha S., Majumder K. (2019). Structural-Features of Food-Derived Bioactive Peptides with Anti-Inflammatory Activity: A Brief Review. J. Food Biochem..

[56] Zhao L., Wang X., Zhang X.-L., Xie Q.-F. (2016). Purification and Identification of Anti-Inflammatory Peptides Derived from Simulated Gastrointestinal Digests of Velvet Antler Protein (Cervus Elaphus Linnaeus). J. Food Drug Anal..

[57] Chakrabarti S., Wu J. (2015). Milk-Derived Tripeptides IPP (Ile-Pro-Pro) and VPP (Val-Pro-Pro) Promote Adipocyte Differentiation and Inhibit Inflammation in 3T3-F442A Cells. PLoS ONE.

[58] Rivera-Jiménez J., Berraquero-García C., Pérez-Gálvez R., García-Moreno P.J., Espejo-Carpio F.J., Guadix A., Guadix E.M. (2022). Peptides and Protein Hydrolysates Exhibiting Anti-Inflammatory Activity: Sources, Structural Features and Modulation Mechanisms. Food Funct..

[59] Jiehui Z., Liuliu M., Haihong X., Yang G., Yingkai J., Lun Z., An Li D.X., Dongsheng Z., Shaohui Z. (2014). Immunomodulating Effects of Casein-Derived Peptides QEPVL and QEPV on Lymphocytes in Vitro and in Vivo. Food Funct..

[60] Tonolo F., Folda A., Scalcon V., Marin O., Bindoli A., Rigobello M.P. (2022). Nrf2-Activating Bioactive Peptides Exert Anti-Inflammatory Activity through Inhibition of the NF-ΚB Pathway. Int. J. Mol. Sci..

[61] Bamdad F., Shin S.H., Suh J.-W., Nimalaratne C., Sunwoo H. (2017). Anti-Inflammatory and Antioxidant Properties of Casein Hydrolysate Produced Using High Hydrostatic Pressure Combined with Proteolytic Enzymes. Molecules.

[62] Baird L., Dinkova-Kostova A.T. (2011). The Cytoprotective Role of the Keap1–Nrf2 Pathway. Arch. Toxicol..

[63] Tonolo F., Coletta S., Fiorese F., Grinzato A., Albanesi M., Folda A., Ferro S., De Mario A., Piazza I., Mammucari C. (2024). Sunflower Seed-Derived Bioactive Peptides Show Antioxidant and Anti-Inflammatory Activity: From in Silico Simulation to the Animal Model. Food Chem..

[64] Chidike Ezeorba T.P., Ezugwu A.L., Chukwuma I.F., Anaduaka E.G., Udenigwe C.C. (2024). Health-Promoting Properties of Bioactive Proteins and Peptides of Garlic (Allium Sativum). Food Chem..

[65] Fan H., Bhullar K.S., Wang Z., Wu J. (2022). Chicken Muscle Protein-Derived Peptide VVHPKESF Reduces TNFα-Induced Inflammation and Oxidative Stress by Suppressing TNFR1 Signaling in Human Vascular Endothelial Cells. Mol. Nutr. Food Res..

[66] Xuan J., Feng W., Wang J., Wang R., Zhang B., Bo L., Chen Z.S., Yang H., Sun L. (2023). Antimicrobial Peptides for Combating Drug-Resistant Bacterial Infections. Drug Resist. Updates.

[67] Govindarajan D.K., Kandaswamy K. (2023). Antimicrobial Peptides: A Small Molecule for Sustainable Healthcare Applications. Med. Microecol..

[68] Moretta A., Scieuzo C., Petrone A.M., Salvia R., Manniello M.D., Franco A., Lucchetti D., Vassallo A., Vogel H., Sgambato A. (2021). Antimicrobial Peptides: A New Hope in Biomedical and Pharmaceutical Fields. Front. Cell Infect. Microbiol..

[69] Liang Q., Liu Z., Liang Z., Zhu C., Li D., Kong Q., Mou H. (2024). Development Strategies and Application of Antimicrobial Peptides as Future Alternatives to In-Feed Antibiotics. Sci. Total Environ..

[70] Assoni L., Milani B., Carvalho M.R., Nepomuceno L.N., Waz N.T., Guerra M.E.S., Converso T.R., Darrieux M. (2020). Resistance Mechanisms to Antimicrobial Peptides in Gram-Positive Bacteria. Front. Microbiol..

[71] Sarkar T., Chetia M., Chatterjee S. (2021). Antimicrobial Peptides and Proteins: From Nature’s Reservoir to the Laboratory and Beyond. Front. Chem..

[72] Brogden K.A. (2005). Antimicrobial Peptides: Pore Formers or Metabolic Inhibitors in Bacteria?. Nat. Rev. Microbiol..

[73] Bechinger B., Gorr S.U. (2017). Antimicrobial Peptides: Mechanisms of Action and Resistance. J. Dent. Res..

[74] Simons A., Alhanout K., Duval R.E. (2020). Bacteriocins, Antimicrobial Peptides from Bacterial Origin: Overview of Their Biology and Their Impact against Multidrug-Resistant Bacteria. Microorganisms.

[75] Grassi L., Maisetta G., Esin S., Batoni G. (2017). Combination Strategies to Enhance the Efficacy of Antimicrobial Peptides against Bacterial Biofilms. Front. Microbiol..

[76] Luo Y., Song Y. (2021). Mechanism of Antimicrobial Peptides: Antimicrobial, Anti-Inflammatory and Antibiofilm Activities. Int. J. Mol. Sci..

[77] León Madrazo A., Quintana Owen P., Pérez Mendoza G., Segura Campos M.R. (2025). Chia Derived Peptides Affecting Bacterial Membrane and DNA: Insights from Staphylococcus Aureus and Escherichia Coli Studies. Plant Foods Hum. Nutr..

[78] Shivanna S.K., Nataraj B.H. (2020). Revisiting Therapeutic and Toxicological Fingerprints of Milk-Derived Bioactive Peptides: An Overview. Food Biosci..

[79] Kulyar M.F.-A., Yao W., Ding Y., Li K., Zhang L., Li A., Waqas M., Huachun P., Quan M., Zeng Z. (2021). Bioactive Potential of Yak’s Milk and Its Products; Pathophysiological and Molecular Role as an Immune Booster in Antibiotic Resistance. Food Biosci..

[80] Wang G., Li X., Wang Z. (2016). APD3: The Antimicrobial Peptide Database as a Tool for Research and Education. Nucleic Acids Res..

[81] Segura-Campos M.R., Salazar-Vega I.M., Chel-Guerrero L.A., Betancur-Ancona D.A. (2013). Biological Potential of Chia (Salvia hispanica L.) Protein Hydrolysates and Their Incorporation into Functional Foods. LWT.

[82] Abadía-García L., Cardador A., Martín del Campo S.T., Arvízu S.M., Castaño-Tostado E., Regalado-González C., García-Almendarez B., Amaya-Llano S.L. (2013). Influence of Probiotic Strains Added to Cottage Cheese on Generation of Potentially Antioxidant Peptides, Anti-Listerial Activity, and Survival of Probiotic Microorganisms in Simulated Gastrointestinal Conditions. Int. Dairy. J..

[83] Wang G., Vaisman I.I., van Hoek M.L. (2022). Machine Learning Prediction of Antimicrobial Peptides. Methods Mol. Biol..

[84] Li T., Ren X., Luo X., Wang Z., Li Z., Luo X., Shen J., Li Y., Yuan D., Nussinov R. (2024). A Foundation Model Identifies Broad-Spectrum Antimicrobial Peptides against Drug-Resistant Bacterial Infection. Nat. Commun..

[85] Asif F., Zaman S.U., Arnab M.K.H., Hasan M., Islam M.M. (2024). Antimicrobial Peptides as Therapeutics: Confronting Delivery Challenges to Optimize Efficacy. Microbe.

[86] Körtvélyessy G., Tarjányi T., Baráth Z.L., Minarovits J., Tóth Z. (2021). Bioactive Coatings for Dental Implants: A Review of Alternative Strategies to Prevent Peri-Implantitis Induced by Anaerobic Bacteria. Anaerobe.

[87] Ferrazzano G.F., D’Ambrosio F., Caruso S., Gatto R., Caruso S. (2023). Bioactive Peptides Derived from Edible Insects: Effects on Human Health and Possible Applications in Dentistry. Nutrients.

[88] Hardan L., Chedid J.C.A., Bourgi R., Cuevas-Suárez C.E., Lukomska-Szymanska M., Tosco V., Monjarás-Ávila A.J., Jabra M., Salloum-Yared F., Kharouf N. (2023). Peptides in Dentistry: A Scoping Review. Bioengineering.

[89] Czarnowski M., Wnorowska U., Łuckiewicz M., Dargiewicz E., Spałek J., Okła S., Sawczuk B., Savage P.B., Bucki R., Piktel E. (2024). Natural Antimicrobial Peptides and Their Synthetic Analogues for Effective Oral Microflora Control and Oral Infection Treatment—The Role of Ceragenins in the Development of New Therapeutic Methods. Pharmaceuticals.

[90] Zhang C., Han Y., Miao L., Yue Z., Xu M., Liu K., Hou J. (2023). Human Β-defensins Are Correlated with the Immune Infiltration and Regulated by Vitamin D3 in Periodontitis. J. Periodontal Res..

[91] Zhao M., Xie Y., Gao W., Li C., Ye Q., Li Y. (2023). Diabetes Mellitus Promotes Susceptibility to Periodontitis—Novel Insight into the Molecular Mechanisms. Front. Endocrinol..

[92] Altalhi A.M., AlNajdi L.N., Al-Harbi S.G., Aldohailan A.M., Al-Ghadeer J.Y., Al-Bahrani J.I., Al-Gahnem Z.J., Alenezi A.H., Al-Majid A. (2024). Laser Therapy Versus Traditional Scaling and Root Planing: A Comparative Review. Cureus.

[93] Khattri S., Kumbargere Nagraj S., Arora A., Eachempati P., Kusum C.K., Bhat K.G., Johnson T.M., Lodi G. (2020). Adjunctive Systemic Antimicrobials for the Non-Surgical Treatment of Periodontitis. Cochrane Database Syst. Rev..

[94] Ramanauskaite E., Machiulskiene V. (2020). Antiseptics as Adjuncts to Scaling and Root Planing in the Treatment of Periodontitis: A Systematic Literature Review. BMC Oral Health.

[95] Mineo S., Takahashi N., Yamada-Hara M., Tsuzuno T., Aoki-Nonaka Y., Tabeta K. (2021). Rice Bran-Derived Protein Fractions Enhance Sulforaphane-Induced Anti-Oxidative Activity in Gingival Epithelial Cells. Arch. Oral Biol..

[96] Tamura H., Maekawa T., Domon H., Hiyoshi T., Yonezawa D., Nagai K., Ochiai A., Taniguchi M., Tabeta K., Maeda T. (2019). Peptides from Rice Endosperm Protein Restrain Periodontal Bone Loss in Mouse Model of Periodontitis. Arch. Oral Biol..

[97] Attik N., Garric X., Bethry A., Subra G., Chevalier C., Bouzouma B., Verdié P., Grosgogeat B., Gritsch K. (2023). Amelogenin-Derived Peptide (ADP-5) Hydrogel for Periodontal Regeneration: An In Vitro Study on Periodontal Cells Cytocompatibility, Remineralization and Inflammatory Profile. J. Funct. Biomater..

[98] Wang H., He H., Cheng X., Feng Q., Yang X., Chen X., Huang Y. (2025). CH02 Peptide-Stimulated Periodontal Ligament Cells Enhance Periodontal Defect Repair in Rats. BMC Oral Health.

[99] Li Y., Ma Y., Yu J., Li C., Yu D., Dai R., Li Q., Cao C.Y. (2023). A Dual Functional Polypeptide with Antibacterial and Anti-Inflammatory Properties for the Treatment of Periodontitis. Int. J. Biol. Macromol..

[100] Wu W., Li G., Dong S., Huihan Chu C., Ma S., Zhang Z., Yuan S., Wu J., Guo Z., Shen Y. (2024). Bomidin Attenuates Inflammation of Periodontal Ligament Stem Cells and Periodontitis in Mice via Inhibiting Ferroptosis. Int. Immunopharmacol..

[101] Kim S.E., Sung H., Shin S., Bae J., Kim G., Lee D., Kim H.W., Seo J., Roh S.Y., Park S. (2025). Evaluation of the Clinical Efficacy of Copine 7-Derived Peptides for Naturally Occurring Periodontitis in Dogs. J. Clin. Periodontol..

[102] Zhang B., Wang L., Liu C. (2024). Expression of TNF-α, Omentin-1, and IL-6 before and after Adjunctive Treatment with a Bioactive Antimicrobial Peptide Periodontal Gel. J. Oral Pathol. Med..

[103] Xiang S., Han N., Xie Y., Du J., Luo Z., Xu J., Liu Y. (2024). Antimicrobial Peptides in Treatment of Stage III Grade B Periodontitis: A Randomized Clinical Trial. Oral Dis..

[104] Wu Y.-F., Han B.-C., Lin W.-Y., Wang S.-Y., Linn T.Y., Hsu H.-W., Wen C.-C., Liu H.-Y., Chen Y.-H., Chang W.-J. (2024). Efficacy of Antimicrobial Peptide P113 Oral Health Care Products on the Reduction of Oral Bacteria Number and Dental Plaque Formation in a Randomized Clinical Assessment. J. Dent. Sci..

[105] Jalali P., Almasi P., Faramarzi M., Hamishehkar H., Kouhsoltani M. (2025). Effect of Spirulina Platensis Algae Purified Bioactive Peptides on Wound Healing after Periodontal Flap Surgery: A Randomized Clinical Trial. Sci. Rep..

[106] Chelliah R., Wei S., Daliri E.B.-M., Elahi F., Yeon S.-J., Tyagi A., Liu S., Madar I.H., Sultan G., Oh D.-H. (2021). The Role of Bioactive Peptides in Diabetes and Obesity. Foods.

[107] Drummond E., Flynn S., Whelan H., Nongonierma A.B., Holton T.A., Robinson A., Egan T., Cagney G., Shields D.C., Gibney E.R. (2018). Casein Hydrolysate with Glycemic Control Properties: Evidence from Cells, Animal Models, and Humans. J. Agric. Food Chem..

[108] Li Y., Fan Y., Liu J., Meng Z., Huang A., Xu F., Wang X. (2023). Identification, Characterization and in Vitro Activity of Hypoglycemic Peptides in Whey Hydrolysates from Rubing Cheese by-Product. Food Res. Int..

[109] Santos-Hernández M., Vivanco-Maroto S.M., Miralles B., Recio I. (2023). Food Peptides as Inducers of CCK and GLP-1 Secretion and GPCRs Involved in Enteroendocrine Cell Signalling. Food Chem..

[110] de Campos Zani S.C., Son M., Bhullar K.S., Chan C.B., Wu J. (2022). IRW (Isoleucine–Arginine–Tryptophan) Improves Glucose Tolerance in High Fat Diet Fed C57BL/6 Mice via Activation of Insulin Signaling and AMPK Pathways in Skeletal Muscle. Biomedicines.

[111] Sharkey S.J., Harnedy-Rothwell P.A., Allsopp P.J., Hollywood L.E., FitzGerald R.J., O’Harte F.P.M. (2020). A Narrative Review of the Anti-Hyperglycemic and Satiating Effects of Fish Protein Hydrolysates and Their Bioactive Peptides. Mol. Nutr. Food Res..

[112] Garcés-Rimón M., Morales D., Miguel-Castro M. (2022). Potential Role of Bioactive Proteins and Peptides Derived from Legumes towards Metabolic Syndrome. Nutrients.

[113] Valenzuela Zamudio F., Segura Campos M.R. (2022). Amaranth, Quinoa and Chia Bioactive Peptides: A Comprehensive Review on Three Ancient Grains and Their Potential Role in Management and Prevention of Type 2 Diabetes. Crit. Rev. Food Sci. Nutr..

[114] Chatterjee C., Gleddie S., Xiao C.-W. (2018). Soybean Bioactive Peptides and Their Functional Properties. Nutrients.

[115] Sanz M., Herrera D., Kebschull M., Chapple I., Jepsen S., Berglundh T., Sculean A., Tonetti M.S. (2020). Treatment of Stage I–III Periodontitis—The EFP S3 Level Clinical Practice Guideline. J. Clin. Periodontol..

[116] American Dental Association. https://www.Ada.Org/Resources/Research/Science/Evidence-Based-Dental-Research/Nonsurgical-Treatment-of-Periodontitis-Guideline.

[117] American Academy of Periodontology. https://www.Perio.Org/for-Patients/Periodontal-Treatments-and-Procedures/Non-Surgical-Treatments.

[118] Tonolo F., Fiorese F., Rilievo G., Grinzato A., Latifidoost Z., Nikdasti A., Cecconello A., Cencini A., Folda A., Arrigoni G. (2025). Bioactive Peptides from Food Waste: New Innovative Bio-Nanocomplexes to Enhance Cellular Uptake and Biological Effects. Food Chem..

[119] Bortoluzzi M. (2025). Created in BioRender. https://BioRender.com/k8h235l.

[120] Silva R. (2025). Created in BioRender. https://BioRender.com/vxirtdy.

